# A systematic mixed methods review: Recovering from a hip fracture in a health promoting perspective

**DOI:** 10.1002/nop2.214

**Published:** 2018-11-18

**Authors:** Johanne Lind, Marianne Mahler

**Affiliations:** ^1^ Department of Nursing, Faculty of Health and Technology University College Metropol Copenhagen N Denmark; ^2^ Department of Education, Learning and Philosophy Aalborg University Copenhagen Copenhagen SV Denmark

**Keywords:** coping, empowerment, health promotion, hip fracture, literature review, mixed methods review, nurses, older adults, recovery, self‐efficacy

## Abstract

**Aim:**

To describe and interpret how older adults who have returned home to recover from a hip fracture cope with life in a health promoting perspective.

**Design:**

Data were collected through the search in seven electronic databases during 2014. Inclusion criteria were peer reviewed, empirical studies. The keywords were hip fracture, coping, empowerment, self‐efficacy, elderly, frail elderly, oldest old. Seventeen papers published 1991–2014 were eligible for inclusion. Analysis of the qualitative and quantitative papers was conducted separately guided by the key words coping, empowerment and self‐efficacy. Due to diversity of studies, a meta‐analysis was not performed. The findings were reported in a narrative synthesis.

**Results:**

Recovery is an individual process of regaining health. It is important to include the person's resources and own goals in life. Physical training must be combined with psychosocial interventions to promote personal engagement and health.

## 1 INTRODUCTION

A hip fracture following a fall is a dramatic event often causing disability and death. Most patients are frail and need medical care and intensive rehabilitation. The mean age is above 80 years, and comorbidity is common (Härstedt, Rogmark, Sutton, Melander, & Fedorowsky, 2015; Hauer, Specht, Schuler, Bärtsch, & Oster, 2002). Hip fracture is a public health issue because of the worldwide ageing population. The entire world is observing a dramatic increase in the population over 60 years (WHO, 2002) and the number of 80‐year‐olds (the oldest old) will triple in Europe from 2008 to 2060.

The incidence of hip fractures is decreasing especially among females due to prevention (Engberg, Curry, & Creswell, 2008; Møller, Damm, & Laursen, 2010), but a higher amount of hip fractures are expected due to the increased elderly population (Nymark, 2008).

In 2006, WHO defined Healthy Ageing as “The process of optimizing opportunities for physical, social and mental health to enable older people to take an active part in society without discrimination and to enjoy an independent and good quality of life” (Swedish National Institute of Public Health R, 2006, p 16). This highlighted the importance of having control over one´s life and having the capacity to define one´s needs.

## 2 BACKGROUND

Gerontology is a research field on old age comprising of disciplines like nursing science, psychology and sociology (Katz, 1996). Gerontological research has both a phenomenological and constructionist perspective on ageing, focusing on finding the meaning in everyday life of older adults (Gubrium & Holstein, 1999; Mahler & Sarvimäki, 2012). Critical gerontology focuses on the pervasive stereotyping of people in later life. Integrating the voices of the older adults in research will develop the debate that can enliven gerontology from becoming complacent (Katz, 2009; Ray, 2008). “Older adults” is in accordance with WHO (2015) and only loosely associated with age in years.

According to WHO (1986), health promotion is the process of enabling people to increase control over and to improve their health. This means that people regardless of age have the right to have control over their lives and be able to cope with stressors in their everyday life to reach a healthy life. To strengthen health promotion, older adults, who have returned to their homes after an operation for a hip fracture, must be able to take active part in decision‐making processes concerning their daily life. Empowerment in healthcare requires that health professionals acknowledge the right of the older adults to self‐determination and that they are enabled to meet this challenge (Berg, Sarvimäki, & Hedelin, 2010). Health professionals must include the home of the older adults as a health promoting arena and support their self‐efficacy (Mahler et al., 2014). Antonovsky (1979, 1987 ) described health promotion as a Sense of Coherence (SOC). SOC contains three dimensions that describe what facilitate individuals´ coping with challenges:* comprehensibility* ‐ the ability to comprehend events to be consistent, predictable and explicable, *manageability* ‐ the capacity to take advantage of and trust resources to deal with challenges and *meaningfulness* ‐ the motivation and commitment to cope with and influence decisions. The Generalized Resistance Resources (GRR) facilitates the individual´s SOC, for example, income, housing, faith and social support.

It is important to do research on how the intentions of the WHO and the theories of health promotion are practiced in the collaboration between the older adults and the health professionals. Likewise, it is important to do it in an intersectional perspective with attention to the meaning of, for example, age, gender, ethnicity and socio‐economic factors (Krekula, Närvänen, & Näsman, 2005). Much literature has been published about home‐dwelling older adults’ recovery from a hip fracture at home. However, little is known about how health promotion is reflected in this literature. This paper reviews published research on how the recovery of home‐dwelling older adults, who have suffered a hip fracture, are described in a health promoting perspective. Thus, the aim of the study was to describe and interpret how older adults who have returned home to recover from a hip fracture cope with life in a health promoting perspective.

### 2.1 Clarification of concepts

#### 2.1.1 Recovery

Recovery is an alternative to the medical orientation often seen in rehabilitation. Recovery includes concepts as hope, self‐determination, empowerment, freedom from stigma and discrimination and the right to a meaningful life (The Department of Health, 2013; WHO, 2017). In everyday life research, recovery is often included in the concept of rehabilitation and the International Classification of Functioning, Disability and Health (ICF) (WHO, 2001).

#### 2.1.2 Coping

In the salutogenic model, Antonovsky (1987) described three concepts: *comprehensibility, manageability* and *meaningfulness* that facilitate the individual's capacity to take up the challenges and maintain health and thereby cope with stressors like a hip fracture. To promote a successful coping with stress, the older adult must have the required resources and perceive the task as challenging.

#### 2.1.3 Empowerment

WHO (2009) defined empowerment as: “A process through which people gain greater control over decisions and actions affecting their health.” In this review, the individual aspect is at stake and empowerment is referred to as psychological empowerment where people have a sense of control over their lives.

#### 2.1.4 Self‐efficacy

Bandura (1977) defined self‐efficacy as the belief that people can perform behaviour necessary to reach their goals. Self‐efficacy determines how people feel, think, motivate themselves and behave.

## 3 THE STUDY

### 3.1 Design

This is a systematic, mixed methods review (Creswell, Klassen, Clark, & Smith, 2011; Sandelowski & Barroso, 2007) including empirical research articles with both a qualitative, quantitative and mixed method methodology. The design integrates findings generated from both different researchers’ approaches and from different participant positions.

### 3.2 Method

In mixed methods, quantitative and qualitative data are integrated to increase the strengths of the validity and to develop a more complete understanding, a complementary picture of a research problem (Creswell et al., 2011). In doing so, the researcher needs to have a pragmatic approach and recognize the existence of both the natural and the physical world and reject the traditional dualisms, that is, rationalism and empiricism (Robson, 2011). Integrative reviews allow for promoting of a holistic analysis and interpretation of the research aim and improving the evidence in nursing (Whittemore & Knafl, 2005).

### 3.3 Search strategy

**Figure 1:**
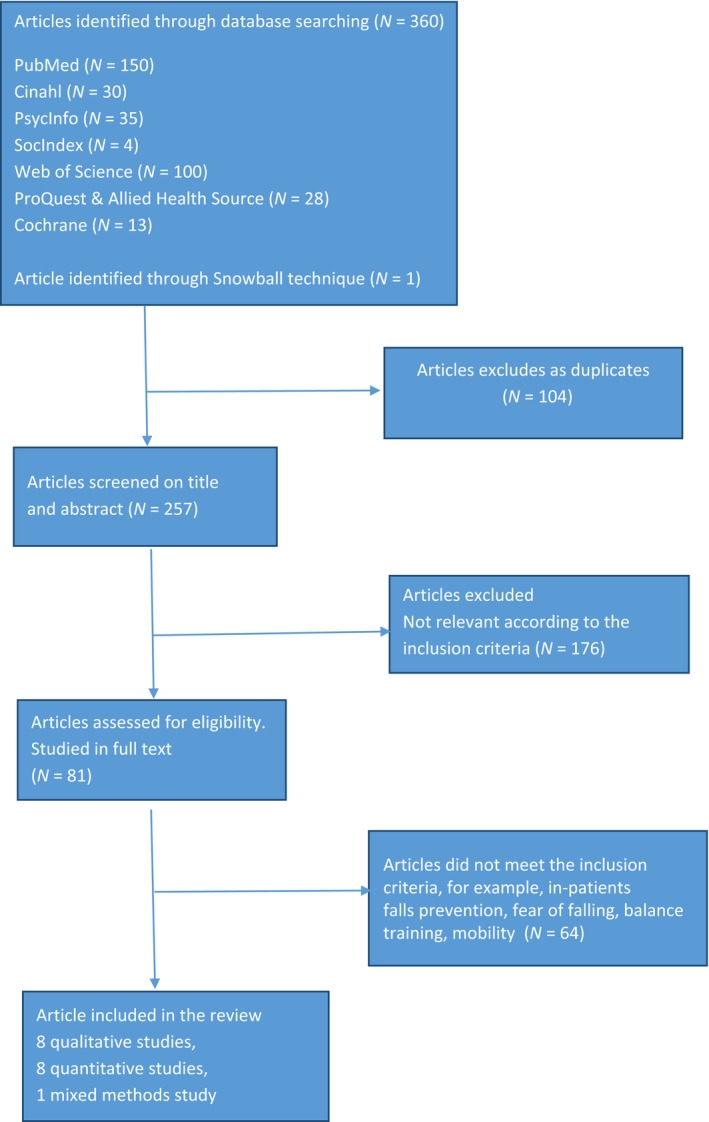
Flow diagram of the data search

A systematic literature search was conducted in 2014 using seven online databases: PubMed, SocIndex, Cinahl, Web of Science, PsycInfo, ProQuest Nursing & Allied Health Source and Cochrane. The databases were searched for both Free Text Words and MESH (Medical Subject Headings) Terms (PubMed and Cochrane), Subject Terms (SocIndex), Headings (Cinahl) and Thesaurus (PsycInfo and ProQuest Nursing & Allied Source). Studies were searched in databases and by using the snowball technique through references of included articles. Search with the term *health promotion* revealed articles about prevention, fear of falling or physical exercise. Therefore, the key words were ((Hip fracture*) AND (coping OR empowerment OR self‐efficacy)) AND (elderly OR frail elderly OR oldest old). Inclusion criteria were original, peer reviewed, empirical qualitative, quantitative or mixed methods studies published in scientific journals. The participants were older adults ≥60 years who had had a hip fracture repair after a fall and had returned to their homes after a hospital and/or rehabilitation stay. Excluded were older adults with dementia or critically ill. Only papers in English were included. The outcome from the database search was 360 studies; 104 duplicates were excluded, and the rest were screened on title and abstract by the first author. After further examinations and discussions between the authors, who are both educated in public health, full text of 81 studies was assessed for eligibility. Seventeen studies were included in the review (Figure 1).

#### 3.3.1 Study designs

Eight studies were qualitative studies. Different designs were represented, for example, qualitative in‐depth interviews—individual or with next of kin, focus group methodology and participant observation generating field notes and including a collection of archival materials (e.g., pictures, diaries; Table 1). Eight studies were quantitative studies with different designs, for example, cross‐sectional studies, a prospective cohort study, a non‐randomized study and randomized controlled trials (Table 2). One study was a mixed methods study with both individual in‐depth interviews and a questionnaire (Table 3).

**Table 1:** Qualitative studies included in the review

Author (year) Country	Aim of the study	Design, sample, response rate, setting	Data analysis, outcomes, instruments	Results
Archibald (2003) England	Reveal participants´ experiences in order to gain insights into how to improve the nursing care of people after hip fracture	In‐depth interviews. Open‐ended, unstructured approach *N* = 5 4 females, 1 male >65 years The interviews took place 5 weeks to 3 months after hip fracture in the participants´ homes	Phenomenological approach	4 main themes: The injury experience Hopeless and helpless, fear The pain experience Most participants could remember pain at time of injury but focused on how they managed The recovery experience Motivation, a key factor in the recovery The disability experience Being housebound, acceptance of new situation
Huang et al. (2014) Taiwan	Explore (a) The ageism perceived by Taiwanese elderly patients during recovery from hip fracture (b) Their feelings and responses to ageism	Observation and semi‐structured interviews. Open‐ended questions *N* = 11 6 females, 5 males ≥ 60 years Health education and exercise suggestions. The interviews took place 1, 3, 6 and 12 months after discharge in the participants’ homes	Content analysis and NVIVO 6.0 software to group words into categories. Key concepts and variables identified as initial codes by deduction from related articles about ageism and defined each category Data saturation	*Perceived ageism: Positive ageism Isolation Neglected *Participants’ responses: Disregard and tolerance of ageism Becoming more independent because of ageism. *Influence of ageism on the lives of elderly hip fracture patients: Reduced autonomy Deprived them of power
Huang and Acton (2009) Taiwan	Explore the ways in which elders who have experienced a hip fracture maintain their independence	Qualitative interviews. One open‐ended question to explore the topic of hip fracture and independence in depth. A questionnaire regarding demographic data *N* = 15 10 females, 5 males ≥ 65 years The interviews took place within 12 months after hip fracture in the participants’ homes	Phenomenological approach. Content analysis. Categories that represented interpretations about ways to maintain independence were identified. Data collection and analysis were simultaneous and iterative	195 statements and 3 major themes exemplified how independence was maintained: Social support Resilience Acceptance of the natural process
Robinson (1999) USA	Identify factors that promote function and enable a successful transition to home for elderly women who are recovering from hip fracture	Focus group interviews. Groups of 7, 5 and 3 participants *N* = 15 All females >70 years Interview guide of open‐ended questions The interviews took place within 9 months after discharge All Caucasians	Grounded Theory approach. Open coding to conceptualize data and axial coding to assemble data and make connections between category and its subcategories	Function‐inhibiting factors Physical discomfort Feeling limited Bending precautions Need for assistive devices Loss of enabling skills Adaptive approaches to life Viewing age as strength Looking ahead—motivation Confronting head‐on—determination, Minimizing problems Seeing humour in frustration Faith—trust in God Function‐promoting factors Recognizing progress Making adaptations for ADLs Accepting help Expressions of well‐being Thankfulness Pride in conquering
Shawler (2006) USA	Describe the concept of Becoming Empowered—a concept in a theoretical framework for transitions in mother–daughter relationships during health crisis	Semi‐structured interviews *N* = 6 older mother–adult daughter dyads The mothers >75 years The daughters <67 years *Individual interviews with mothers and daughters *Mother and her daughter were interviewed together *2 focused discussion groups with 3 mother–daughter dyads in each group *Participant observation of the mothers and their daughters in their own homes The interviews were completed in either the participants’ homes or in a private dining room of a local café All Caucasian	Grounded Theory approach Data collection and analysis was cyclic and recursive. Successive interview was coded and compared and contrasted with previous data	A theoretical model, The Evolutionary Empowerment‐Strength Model about the transitions experienced by the mothers and daughters was developed Modelling courage Mothers and daughters demonstrated mental strength to persevere and withstand fearful experiences, served as exemplars to others and grew from challenging and fearful times Sharing ideas and empowering ways Sharing specific ideas about how to strengthen oneself during and after a crisis, a sense of self‐confidence. Offered empowerment in decision‐making and seeking information. Offered advice about physical activity, shared ideas and beliefs in divine power and their life philosophies
Shawler (2007) USA	Discover the individual women's perspectives of their relationships over time while going through a health crisis	Semi‐structured interviews *N* = 6 older mother–adult daughter dyads The mothers >75 years The daughters <67 years *Individual interviews with mothers and daughters *Mother and her daughter were interviewed together *2 focused discussion groups with 3 mother–daughter dyads in each group *Participants’ archival materials (e.g., photographs, poetry, sewing projects) The interviews were completed in the participants’ homes or in a private dining room of a local café and lasted 2 hr All Caucasian	Grounded Theory approach Data gathering was guided by the concepts emerging from the theory. Constant comparative method enhanced theoretical sampling and involved continually reviewing and analysing the data, re‐interviewing individuals to obtain participant validation and collecting data to support themes of the emergent theory	Evolution of Women's Strength. It answers the question of what basic social psychological process helps the women move through the health crisis *Internalizing female lineage—heritage, ancestral qualities, sources of strength *Mastering maturity—responsibility, higher levels of wisdom *Evolving matriarchs—used strength to manage intergenerational changes in traditions *Emerging transformation—incorporating joyful surprises and difficulties into their life perspectives *Becoming empowered—by modelling courage and sharing advice Evolution is a “process of continuous change form a lower, simpler or worse state to a higher more complex or better state: Growth”
Young and Resnick (2009) USA	Determine why some hip fracture survivors return to their pre‐fracture function and others do not, this article explores factors associated with functional recovery from the patient's perspective	A thematic interview with open‐ended questions exploring areas influencing functional recovery and participants’ willingness to engage in rehabilitation activities *N* = 62 ≥ 65 years 76% females 92% caucasians Convenience sampling, Part of a larger longitudinal study The interview took place 12 months after hip fracture	Content analysis. Interview guide. The analysis was consistent with in vivo coding Codes were grouped. Twenty‐five codes were identified and collapsed into four major themes	Facilitators of the recovery Professional care Social support and spirituality Determination Lifestyle factors and environment Identifying goals Factors that hindered recovery Medical complications/comorbidities Unpleasant sensations (pain) System recommendations to facilitate recovery Increase the amount of care provided Spirituality/Social support Peer advice and recovery Participate and listen to healthcare providers Determination and a positive attitude Be careful Relieve pain or work through pain
Zidén et al. (2008) Sweden	Explore and describe the experienced consequences of an acute hip fracture among home‐dwelling elderly people shortly after discharge from hospital	Qualitative interviews. The participants narrated their experiences *N* = 18 16 females 2 males ≥ 65 years Recruited from a larger sample of 102 participants in an intervention study for people with acute hip fracture (Zidén et al., 2010) The interviews took place 1 month after discharge in their homes	Analysed according to the phenomenographic method A computer program, Open Code 2001, was used to facilitate the sorting of the interviews and the quotations	*In relation to your body and to yourself: Limited to move and lost confidence in body Become humble and grateful Respect yourself and your own needs *In relation to others: Become more dependent on others Gain more human contact and are treated in a friendly way by others *In relation to the life situation: Secluded and trapped at home You are old, closer to death and Have lost your zest for life You take one day at a time and are uncertain about the future

**Table 2:** Quantitative studies included in the review

Authors (year) Country	Aim of the study	Design, sample, response rate, setting	Data analysis, outcomes, instruments	Results
Casado et al. (2009) USA	Hypothesized that social support for exercise from experts (*SSE*‐E) would directly and indirectly influence exercise behaviour through their self‐efficacy and outcome expectations for exercise	Prospective, descriptive study. Secondary analysis of data from a RCT study *N* = 164 All females ≥ 65 years Face‐to‐face interview 2 and 6 months post‐hip fracture in the participants homes All Caucasian	Comparisons between groups The Yale Physical Activity Survey (YPAS) The Outcome Expectation for Exercise (OEE) Scale The Self‐Efficacy for Exercise (SEE) Scale The Social Support for Exercise Habits (SSEH) Scale SF−36	The Social Support for Exercise Habits Experts Scale (SSEH‐E) increased over time in all groups. Statistically significant increase in social support for exercise between participants in the control versus any of the treatment groups. Those who were exercising with a trainer and receiving the motivational Plus component had stronger social support for exercise and outcome expectations for exercise
Fortinsky et al. (2002) USA	Test the hypothesis that hospitalized hip fracture patients with greater reported self‐efficacy for conducting rehabilitation therapy would have a greater likelihood of recovering to pre‐fracture levels physical function 6 months after the fracture	A prospective cohort design *N* = 41 patients consented 34 completed self‐efficacy data 24 completed 6 months follow‐up data Among the 41 patients +15 proxies 82% were females Mean age 82 years. All but one Caucasians Interviewed about pre‐fracture function 4 weeks prior to the hip fracture, current level of self‐efficacy for rehabilitation therapy, current level of depressive symptoms and other measures of physical and mental health Six months after the fracture, a telephone interview was conducted with study patients or proxies to determine patients’ current level of functioning	Descriptive statistics to inspect the distribution of responses to eight rehabilitation therapy self‐efficacy items Logistic regression analysis to determine whether the likelihood of locomotion recovery was associated with rehabilitation therapy self‐efficacy Evaluation of the independent effect of rehabilitation self‐efficacy on locomotion recovery after adding the level of depressive symptoms as a covariate Center for Epidemiological Studies‐Depression Scale (CES‐D) The physical functional independence measure (FIM)	Results showed that patients with higher self‐efficacy scores had a greater likelihood of locomotion recovery, controlling for pre‐fracture locomotion function level This positive association between rehabilitation therapy self‐efficacy and likelihood of locomotion recovery persisted after adding depressive symptoms to this logistic regression model. Depressive symptoms were not found to be associated with likelihood of functional recovery
Johansson et al. (1998) Sweden	Explore the predictive power of SOC in patients with hip fractures in reference to length of hospital stay, pre‐ and postoperative state of confusion, health, functional ability, quality of life, and need for help after discharge form hospital	A non‐randomized intervention study 73 patients—during the study reduced with 8 patients. ≥ 60 years 77% were females 32 patients from particular districts were involved in an intervention programme aimed at improving the rehabilitation process. The control group lived in other districts One and four months after discharge quality of life scores were almost identical, and the analysis and results are therefore based on the entire group	Dichotomizing the SOC subgroups, means and proportions between the high and low SOC SOC was measured with a scale developed by Setterlind and Larsson QoL was measured by the QoL Index (QLI) The Standardised Practical Equipment (SPE test) tested the activities of daily life Complementary questions concerning the person´s dependence	One month after discharge: persons with stronger SOC—more had returned home, less help from the municipality, expressed less disability and fewer experiences of discomfort regarding emotional status Four months after discharge persons with stronger SOC had higher scores (favourable) on all subscales of the SPE test, the overall QLI, and the four subscales of the QLI The patients with a weaker SOC were significantly more dependent on assistance in social activities and needed more help from the municipal services
Pakkala et al. (2012) Finland	Describe the effects of intensive resistance training on SOC among older adults after hip fracture	Secondary analyses of a randomized controlled trial. Design and method published previously (Portegijs et al. 2014) *N* = 46 Intervention: 8 males and 16 females Control: 6 males and 16 females 60–85 years Intervention: a 12‐week individually tailored, strength‐power training programme, twice a week 0.5 to 7 years after hip fracture	Antonovsky´ s. 13‐item scale The Yale Physical Activity Survey (YPAS) The Visual Analog Scale (VAS)	Intensive 12‐week strength‐power training had no significant effect on participants' SOC level In both groups SOC scores decreased, but the change was non‐significant
Portegijs et al. (2014) Finland	Determine the relationship between SOC and (a) training adherence (b) changes in muscle strength, mobility, and balance in older people with a history of hip fracture	Secondary analyses of a randomized controlled trial. Design and method published previously (Portegijs et al. 2014) *N* = 45 Intervention: 8 males and 15 females Control: 6 males and 16 females 60–85 years Intervention: 12 weeks of resistance training 0.5. to 7 years after hip fracture	SOC Antonovsky’ s short 13‐item scale Isometric knee extension torque. Mobility test maximal speed The modified timed up and go (TUG) Berg Balance Scale (BBS)	SOC was significant associated with adherence No significant association between maximal walking speed and knee extension torque TUG showed a significant association with SOC, but no associations were found for BBS
Roberto (1992) USA	Investigate the nature of strategies used by older women to cope with their hip fractures	Cross‐sectional study Structured interview and review of the participants´ medical record *N* = 101 All females ≥ 65 years All Caucasian Demographic information, questions about functional abilities and psychosocial variables related to the recovery process The interview took place in the respondents´ s homes 8 months after their hip fracture	*Personal variables: Chronic illnesses and tasks of daily living (OARS), cognitive functioning, locus of control orientation (Locus of control Scale) and network size *Material resources: Categories of income was counted, coping strategies (The Ways of Coping Questionnaire), *Perceived recovery: physical functioning was rated by the women, psychological functioning (The Beck Depression Inventory)	Seeking social support was the most common strategy Women with a high chance orientation may be more likely to use distancing as a coping strategy, because they accept their hip fractures as part of the ageing process and out of their control Except for locus of control, none of the other personal and material resources significantly influenced the coping strategies used by the women Several types of coping were associated with the women's perceived recovery. The use of self‐controlling, accepting responsibility, escape‐avoidance and positive reappraisal was negatively associated with perceived physical functioning. These strategies reduced the distress
Shaw et al. (2003) England	Assess the relationship between health locus of control and outcome in older women who had undergone surgery for fractured neck of femur	Descriptive questionnaire survey The data for this study were collected as a part of a larger study (no reference) *N* = 112 completed interview 1 and 2 All females ≥ 64 years Interview at 5 and 30 days post‐surgery at hospital and at home respectively	To assess psychological distress, The Hospital Anxiety and Depression Scale (HADS) was used To assess functional ability, the frequency of required help in ADL was measured To measure control beliefs in relation to recovery and rehabilitation The Recovery Locus of Control scale	Increase in measures between 5 days and 30 days follow‐up in depression, levels of disability and a little in anxiety Higher score on the locus of control scale was associated with less disability and greater independence in daily living Relations between locus of control and anxiety at 30 days measured tended to be associated
Zidén et al. (2010) Sweden	Describe the long‐term effects of a home rehabilitation programme in elderly people with hip fracture compared with conventional care regarding independence in daily activities, balance confidence, frequency of daily activities, physical functioning, HRQoL and perceived recovery	A randomized controlled trial. Geriatric hospital‐based home rehabilitation was compared with conventional care *N* = 102 Intervention: 29 females, 19 males Control: 42 females, 12 males ≥65 years Conventional Care: Standard care, discharged to their own home with no further organized rehabilitation Home rehabilitation (HR): First: Specially individually designed early goal‐setting with the patient. Prepared and supported discharge, close cooperation with relatives and social home services aimed at mobilizing the person´s motivation and self‐efficacy Second: 3 weeks with home visits by the same physiotherapists, occupational therapists and nurses Discharge measurements and follow‐up data 6 and 12 months were collected via face‐to‐face interview or by telephone	Independence in activities of daily living (FIM) Independence in instrumental daily activities (IAM) Frequency of activity, The Frenchay´s Activity Index (FAI) Basic physical mobility, The timed up and go (TUG) Functional lower extremity muscle strength, Sit to Stand (STS) Balance confidence, Falls Efficacy Scale, Swedish version (FES (S)) Health related quality of life (SF−36) Mood, The Centre for Epidemiological Depression Scale (CES‐D) Participant characteristics, walking habits and perceived recovery were measured 6 and 12 months after discharge	12 months post‐discharge the HR participants reported significantly higher degree of independence in self‐care, locomotion (FIM) and outdoor activities Frequency of activities (FAI) showed higher degree in total score and outdoor activities both after 6 and 12 months The HR participants reported higher degree of confidence in performing activities without falling (FES(S)) On the SF−36 scale participants reported significantly higher degree of physical activity and less Body Pain. No other data from SF−36 were shown The participants did not show any differences in depression One year after discharge 14 persons (29%) in the HR group and five persons (9%) in the CC group considered themselves fully recovered

**Table 3:** Mixed methods study included in the review

Authors (year) Country	Aim of the study	Design, sample, response rate	Data analysis, outcomes, instruments	Results
Borkan et al. (1991) USA	Examine *injury narratives*. Research questions: What are the meanings present in the narratives of elderly hip fracture patients? What are the importance of narrative elements as prognostic indicators or “risk factors” for predicting rehabilitation outcomes?	Individual, in‐depth interviews during the initial hospital admission, field notes, a functional status questionnaire, follow‐up interviews at 3 and 6 months post‐hip fracture in the participants homes or by telephone on request *N* = 80, 81% females >65 years	Qualitative narrative and quantitative analysis Questionnaire included of measure functional status, activities of daily living (ADL), social function, psychological well‐being and sickness behaviour Systematic quantitative analysis of narratives focused on the relationships between narrative scores form the interviews and the changes in ambulation All dimensions were analysed both singly and in terms of their composite variables	The relationship between narrative analysis and outcomes—the actual outcomes for analysis were change in ambulation from pre‐fracture level to 3 and 6 months post‐fracture (ADL scale) The participants who explained the physical event within a mechanistic model did better with regard to change in ambulation The participants who perceived the fall as organic cause showed greater improvement in self‐confidence and an optimistic approach The participants who perceived the impact of their fracture as having little or no influence on their autonomy showed much greater improvement in ambulation Most subjects reflected a mechanistic world view

#### 3.3.2 Intersectional comments

The majority of the participants were European or American. Participants in two studies were from Taiwan. There was limited information concerning cultural background such as gender, race and religion. The majority of participants lived alone. Most of the studies did not specify the economic situation of the participants. The educational background varied from college degrees to illiterate participants. The authors mostly characterized their participants’ background as *different* or *varied*.

### 3.4 Quality appraisal

The methodological quality of the included studies was critically assessed. The qualitative studies were systematically examined to assess their validity by using the basic criteria for validity of qualitative studies inspired by Lincoln and Guba (1985) (Table 4). The quantitative studies and the mixed methods study were assessed following (The Critical Appraisal Skills Program, 2013; Taylor, Reeves, Ewings, & Taylor, 2004) (Table 5).

**Table 4:** Appraisal of the qualitative studies in the review inspired by Lincoln and Guba (1985)

	Archibald (2003)	Huang et al. (2014)	Huang and Acton (2014)		Robinson (1999)	Shawler (2006)	Shawler (2007)	Young and Resnick (2009)	Zidén et al. (2008)
Credibility	X No experienced co‐author	X	X	X		X No experienced co‐author	X	X	X
Transferability	X	X	X	X		X	X	X	X
Dependability	X	X	X	X		X	X	X	X
Confirmability	X	X	X	X		X	X	X	X
Data saturation		X	X			X	X		
Ethical considerations	X	X	X	X		X	X	X	X

In all included studies, researchers did reflections on credibility (e.g., intern validity, guidance by an experienced researcher), transferability (e.g., external validity, strategic sampling), dependability (e.g., reliability, the process of the research is logical, traceable and clearly documented), confirmability (e.g., transparence, findings are clearly derived from the data, discussed pre‐understanding), and they all showed ethical considerations. Four studies observed data saturation.

**Table 5:** Validity of the quantitative studies and the mixed methods study in the review inspired by Critical Appraisal Skills Program (CASP)

	Casado et al. (2009)	Fortinsky et al. (2002)	Johansson et al. (1998)	Pakkala et al. (2012)	Portegijs et al. (2014)	Roberto (1992)	Shaw et al. (2003)	Zidén et al. (2010)	Borkan et al. (1991)
Design	X	X	X	X	X	X	X	X	X
Sample	X	X	X	X	X	X	X	X	X
Analysis	X	X	X	X	X	X	X	X	X
Bias discussed	X	X	X	X	X	X	X	X	X
Confounder	Well educated, Caucasian females, not accounted for participants former behaviour	Small sample size only about half fulfilled the study, younger, more independent	X	Small sample size, healthy and young participants, relatively high SOC	Small sample size, Participants with high SOC, follow‐up period 3 months, that is, too short to detect. Differences in SOC, measured 0.5–7 years after hip fracture	X	Participants made a retrospective judgement of pre‐fracture status, measure Short time after hip fracture	Participants in the conventional care group were older, more males, had more help at home. The intervention group had early start in rehabilitation, 44 participants were excluded after randomization	X
Randomization	Explained in a former study	Not relevant	Not relevant	Secondary analysis of RCT	Published previously	Not relevant	Not relevant	X	Not relevant
Ethical considerations	X	X	X	X	X	X	X	X	X

### 3.5 Data abstraction and synthesis

Data from the included studies were abstracted to facilitate the review. Characteristics from the studies were established in matrices, that is, author, publication year and country, aim, design, sample, response rate, setting, analysis, outcomes, instruments and results (Tables 1–2). This emphasized equal value between the methodologies and a preliminary interpretation of patterns and relationships within and across the papers became visible (Whittemore & Knafl, 2005).

The key words coping, empowerment and self‐efficacy guided the analysis and findings were summarized and presented under separate headings. As the included studies represent great methodological diversities and outcome measures, it was not possible to conduct a meta‐analysis but more appropriate to report the findings in a narrative way. In the discussion, the results were integrated.

### 3.6 Ethics

As this study was a systematic mixed method review, ethical approval was not required.

## 4 RESULTS

### 4.1 Qualitative studies

The eight qualitative studies included 110 women and 28 men who had returned home after an operation for a hip fracture.

#### 4.1.1 Coping

The coping strategies were characterized by a battle for independence (Huang & Acton, 2009), active participation and willingness to engage in their recovery (Young & Resnick, 2009). Zidén, Wenestam, and Hansson‐Scherman (2008) found that individuals with “limitations in movement and being dependent on others” were less able to cope. They described their experience of a hip fracture with the words “lost confidence in your body,” “I think it's meaningless, what I do.” In spite of these statements, the same persons also stated that they had positive life experiences like:

I don´t hung up on small things …. I´ve gotten a perspective on life. I´ve learned to be grateful …. I think you learn things all your life. Because, in spite of everything, I´m health. (Zidén et al., 2008, p. 805)

Despite their apparently difficult conditions, they were able to both comprehend and manage physical and existential hardships. Huang, Liang, and Shyu (2014) described an experience of discrimination because of high age that was a threat to independence and decreasing of autonomy:

Last month, we had a family party. I asked my son to take me out for a haircut and to buy a new shirt, but my son was too busy to promise me. He said, “You look OK. At the party, you only need to sit and eat. No one will notice what you are wearing or what your hair looks like.” (Huang et al., 2014, p. 33)

Oppression and discrimination seem, however, to encourage these older adults to engage more in their recovery and perceive recovery as a challenging task. Following cultural traditions, older Taiwanese would be more patient and accept arrangements made for them, but because the participants perceived their situation as result of an accident and not as result of disease or old age, they actively engaged in their recovery (Huang et al., 2014).

The participants also showed comprehensibility related to advice from others about recommended healthy lifestyle activities to cope with life after a hip fracture. They showed manageability in avoiding a new fall, for example, to furnish their home in a falls‐preventing way (Young & Resnick, 2009).

#### 4.1.2 Empowerment

The participants described their transition as a personal growth opportunity (Archibald, 2003). They had managed difficult times earlier in life and understood the importance of the continued physical workout (Robinson, 1999). These experiences had facilitated their motivation and determination:

“…you´ve got to do the best you can. Even at my age, you catch yourself planning what you are going to do next summer” (Robinson, 1999, p. 1344)

Their recovery approach was characterized by humour, faith, pride and a strength to minimize adversity resulting in power to anticipate the future (Robinson, 1999). Interviews with older women and their daughters (Shawler, 2006, 2007 ) revealed power and mental resources to deal with the experience of a hip fracture by acting as an example to others showing how they “managed, survived and grew from challenging and fearful times” (Shawler, 2006, p. 372). This kind of empowerment was explained with a female lineage through generations of powerful female ancestors (Shawler, 2007). It was a time of promoting skills of learning, growing and adaptation which they succeeded in because of their self‐determination and ability to fight for their demands to be met.

#### 4.1.3 Self‐efficacy

Participants stressed the positive effect the professionals, family and friends had had on their recovery especially the verbal encouragement had lifted their spirits “The help, encouragement and support that I got from my family and friends are essential…” (Young & Resnick, 2009, p. 113). They would have liked more and a better physical therapy and nursing care at home, that is, professionals who could give education in the recovery process. Archibald (2003) stated that motivation to recover was a key aspect in recovery. Some participants showed great enthusiasm for giving good advice to others “Get up and do as much as you can and don´t worry,” “Be positive” (Young & Resnick, 2009, p. 115). Huang et al. (2014) found that perceived ageism could have the reverse effect and thus increase self‐efficacy and facilitate return to their usual capacity in their life pre‐fracture. Despite the experience of physical and psychosocial defeat, the informants showed an irrepressible belief in their ability to overcome barriers and life crisis to recovery and to restore their well‐being.

### 4.2 Quantitative studies

The eight quantitative studies included 584 women and 79 men who had returned home after an operation for a hip fracture.

#### 4.2.1 Coping

Five studies described areas related to managing distress and coping strategies (Johansson, Larsson, & Hamrin, 1998; Pakkala et al., 2012; Portegijs et al., 2014; Roberto, 1992; Shaw, McColl, & Bond, 2003).

Roberto (1992) measured the coping strategies among 101 females and found that seeing the fracture as a part of an ageing process and as such out of control for the individual and with loss of comprehensibility was significantly associated with a distancing strategy (*p* < 0.01) and a high belief in being controlled by powerful others. In another descriptive study of 112 females, Shaw et al. (2003) showed that a greater Internal Locus of Control was significant associated with greater independence in daily living. Three studies measured the association between SOC and recovery from a hip fracture (Johansson et al., 1998; Pakkala et al., 2012; Portegijs et al., 2014). Johansson et al. (1998) found that 4 months after a hip fracture, patients with a stronger SOC had significantly better scores on the quality of life Index, for example, physical health, marriage, family, friendship, stress (*p* < 0.001) and on all subscales of the Standardized Practical Equipment test, for example, ADL, balance and mobility. In addition, they found that patients with a weaker SOC showed a significant higher dependence on assistance in social activities (*p* < 0.01) and help from the municipal services (*p* < 0.001).

The association between SOC and both physical training and muscle strength was measured in two studies originating from the same RCT (Pakkala et al., 2012; Portegijs et al., 2014); 12 weeks of individually tailored, intensive strength training in the intervention group of 24 patients did not improve SOC or any of its components, comprehensibility, manageability and meaningfulness compared with a control group of the similar size (Pakkala et al., 2012). In the study conducted by Portegijs et al. (2014), the association between SOC and adherence to training, changes in muscle strength, mobility and balance was measured. The study showed that all participants improved in muscle strength regardless of their strength of SOC. The analysis revealed, however, that participants with stronger SOC showed a significant positive training adherence (*p* = 0.009) and better coping with their recovery process as they improved in independence in daily life, quality of life, ADL, balance and mobility. Having a stronger SOC meant that they could identify and use their resources and manage complicated physical tasks.

#### 4.2.2 Empowerment

One quantitative study (Zidén, Kreuter, & Frändin, 2010) had an empowerment promotional approach. In a RCT study with 102 participants, the focus was on how an individually designed home rehabilitation (HR) programme would improve daily activities, balance confidence, physical functioning, health related quality of life and perceived recovery. The design included an early individual goal setting with the patients, carefully prepared and supported discharge and close cooperation with relatives and social home services. The main intervention was multi‐professional through several home visits by physiotherapists and occupational therapists and for some visits from nurses and assistant nurses for 3 weeks. Six months post‐discharge, the HR participants reported significantly higher degree of independence in self‐care (*p* = 0.001), locomotion (*p* = 0.012) and outdoor activities (*p* = 0.012), but some of the differences in the groups disappeared at 12 months post‐discharge. Health related quality of life was only shown in results of physically functioning and bodily pain. After 12 months, physically functioning (*p* = 0.001) and bodily pain (*p* = 0.042) remained statistically significant.

The HR thus showed that an intensive support at home in their own setting would mobilize personal resources and enhance the participants’ self‐capacity and ability to participate in self‐care and thus restore the control over their lives.

#### 4.2.3 Self‐efficacy

Two quantitative studies explicitly analysed self‐efficacy in relation to recovery after a hip fracture. Casado et al. (2009) measured whether social support for exercise from experts would influence exercise behaviour in 164 females after a hip fracture. The Social Support for Exercise Habits Scale (SSEH) increased in all groups but with a statistically significant higher increase in the three intervention groups than in the control group (*p* = 0.002). The strongest social support for exercise was seen in the intervention group, exercising with a trainer and receiving motivational support. They also reported stronger outcome expectations for exercise, but there were no reports on stronger exercise behaviour. Fortinsky et al. (2002) measured rehabilitation therapy self‐efficacy using a questionnaire developed for this study; 32%–41% answered the most positive answer for each item. Logistic regression analysis showed a tendency that those with higher self‐efficacy scores had greater locomotion recovery, measured by using the functional independence measure (FIM) (Adjusted OR = 1.21; 95% (CI) = [1.00–1.45]; *p* = 0.05). The findings in the studies showed that receiving support in physical training and verbal persuasion was important but not enough for the participants to change behaviour.

### 4.3 Mixed methods study

The search in the databases revealed one mixed method study that fulfilled the inclusion criteria (Borkan, Quirk, & Sullivan, 1991). It included 65 women and 15 men who had returned home after an operation for a hip fracture. The study had a convergent design (Fetters, Curry, & Creswell, 2013) integrating at the method level—quantitative survey and qualitative interviews. Interviews with both open‐ended questions and standardized scales were conducted with 80 patients during hospitalization and after 3 and 6 months. The analysis sought to gain knowledge about how patients created meanings from an episode of sickness and formulated plans for action, their perception of disability and how they could be perceived in the future.

#### 4.3.1 Coping

The study showed that those patients who explained the fracture to be a result of external cause or an acute incident like a fall coped better with regard to ambulation “I´ll be up and around before you know it.” Whereas those who thought they were sufferers of diseases and believed the fracture was a result of ageing would think of themselves as dependent on others. “I don´t think I´ll ever walk normally again” (Borkan et al., 1991, p. 951). The recovery potential was closely linked to comprehensibility, manageability and meaningfulness.

#### 4.3.2 Empowerment

Perception of disability included evaluation of several dimensions like vulnerability and perception of dependence. Those patients who perceived the fracture to be of little or no influence on their autonomy or independence showed much greater improvement in ambulation. “I´ll get around visiting people” compared with the opposite pole “Nobody seems to be coming to visit me so I don´t know who I belong to anymore” (Borkan et al., 1991, p. 952). Depending on the understanding of how the fracture would influence everyday life the participants showed possibilities for exerting control over decisions and actions in their lives.

#### 4.3.3 Self‐efficacy

The dimension of futurity was evaluated in either hopefulness or hopelessness. Hopefulness was expressed as a belief in full recovery “I´m the type of guy who won´t give in. Stubborn.” Hopelessness was expressed as absence of expectations for recovery “I have nothing to look forward to and I´ll lay here till I die.” (Borkan et al., 1991, p. 953). The degree of autonomy and independence determined participants’ skills and energy to motivate themselves to overcome challenges in everyday life and reach their goals.

## 5 DISCUSSION

This is a review of both qualitative, quantitative and mixed methods peer‐reviewed studies about how older adults who have returned home to recover from a hip fracture cope with life in a health promoting perspective. The findings are described using the three concepts: *coping, empowerment and self‐efficacy, which are central in the health promotion* terminology inspired by the Ottawa Charter (WHO, 1986) and a part of the understanding of SOC. Various approaches to the key concepts were seen, but the studies did not include any clear definitions. The participants´ GRR, which are a fundamental part of health promotion, were not part of the analysis in the studies in this review. This deficiency can be a reflection of the authors’ professional, theoretical and philosophical competences or a sign of silent theoretical knowledge. In this review, WHO (1986) was used as point of reference. Key concepts were illuminated from definitions and texts from WHO.

### 5.1 Coping

*Coping* played an important role after a hip fracture to live a life with physical, social and mental health. The participants’ capacity to cope with life after hip fracture was understood with the concepts of SOC: *comprehensibility, manageability* and *meaningfulness*. The participants emphasized the importance of their personal engagement to live their lives in accordance with their life goals and by following recommendations from professionals. They managed to reverse adversities and tough living conditions for a life of acceptance and active participation. A strong SOC helped to improve physical capacity and quality of life. Meaningfulness was seen by the commitment and engagement where they participated in their recovery (Antonovsky, 1987). A strong SOC might be explained by age and by a natural selection—healthy older adults live longer—or because older adults develop a strong SOC (Eriksson & Lindström, 2005).

It appears in both qualitative and quantitative studies and the mixed method study that the participants’ understanding of the determinants for hip fracture affected their way to cope. Hip fracture can be understood as an acute event, for example, a fall and therefore has to be mastered actively. Alternatively, hip fracture can be understood due to a high age and frailty and therefore a negative experience to which one finds an avoiding coping strategy. To be perceived as dependent and frail is a stereotypic depiction of older adults, which might break down their social and personal identities and contribute to ageism (Angus & Reeve, 2006). Ageist attitudes are devastating threats to ageing well. Age discrimination may lead to chronic stress and diseases (Allen, 2016) and thus act as a barrier to increased control and improvement of health (WHO, 1986).

### 5.2 Empowerment

The participants used different ways to maintain empowerment. In the qualitative studies and the mixed method study, empowerment was a concept closely related to the participants’ personal growth. It was a way of exerting self‐management to become familiar with their changed life conditions. Self‐management of chronic illness is not only “doing” but also “being” and “becoming” and is a continuous development of both the physical and psychosocial capabilities to create order in life (Kralik, Koch, Price, & Howard, 2004).

In the intervention studies, the participants´ received intensive physical training to enhance their empowerment through improved physical mobility. Since the Ottawa Charter (WHO, 1986), the discourse concerning empowerment and patient participation has been dominant among nurses and other health professionals to enhance the autonomy of the patients. Professionals´ instructions in physical exercise in the studies can be perceived as a prescribed behaviour exclusively originating from a professional mindset (Freire, 1993). If health professionals continue to define the goals of the recovery efforts and consider the older adults to be passive recipients, there are only small chances for empowerment and recovery (Hage & Lorensen, 2005).

### 5.3 Self‐efficacy

To master challenges after life threatening events like a hip fracture was a highly demanding task and a continuous battle. The older adults in this study mobilized an inner strength to continue their battle for healthy ageing and autonomy. In their study of inner strength Nygren, Norberg, and Lundman (2007) found inner strength to be engagement in life, responsibility for oneself and others, feelings of pride, having trust in oneself and displaying a willpower. The ready‐made exercise programmes were not enough to change behaviour. Personal recovery is a unique process of changing attitudes, values, goals and roles related to the individual's lifeworld (Slade, Amering, & Oades, 2008). Thus only focusing on symptoms and symptom reduction equals a view on the older adults as passive, maybe compliant recipients belonging to a homogeneous group and not a person that takes active part in their recovery. Perceived self‐efficacy is essential for the coping effort that people will put into recovery. To develop self‐efficacy, people must be supported in identifying their personal experiences and show commitment to their recovery (Bandura, 1977). Professionals must shift the glance from prevention to health promotion, include the individuals´ life goals, work in partnership and respect their autonomy. In a health promotion approach, the role of the professionals is not to dictate tasks but to facilitate possibilities.

### 5.4 Limitations and strengths

This is to our knowledge the first systematic mixed methods review on health promotion and older adults after a hip fracture. All studies that fulfilled the inclusion criteria were included. Some quantitative studies did not include enough patients to do generalization and the qualitative studies originated from very different cultures. The coping resources might be explained by a biased selection of especially resourceful participants. However, the study is valuable due to mixed methods design in its comprehensive investigation of health promotion and recovery. The analysis of the studies was made credible through quotations from the participants, the presentation of significant findings and the detailed explanations of the results in the text and in the Tables.

## 6 CONCLUSION

The findings of this systematic mixed methods review provided evidence for coping, empowerment and self‐efficacy to be important concepts in the study of recovery in a health promoting perspective. The review revealed that promoting coping capabilities must include both physical functions and an emphasized support of the older adults in their individual way to health promotion. To improve recovery comprehensibility, manageability and meaningfulness must be strengthened. Self‐management can improve the participants’ empowerment and self‐efficacy. The experiences of personal growth and trust are essential. Physical training is important but need to be incorporated in the recovery on the participants’ premises in respect of their autonomy and life goals.

Given the increase in the older population worldwide, there is a growing need for research in the complex concepts of health promotion and everyday life of older adults. Research should reveal how autonomy and participation in recovery is promoted and respected. Researchers must be aware of intersectional variations concerning, for example, age, gender, culture, religion and socio‐economic status in research in recovery after hip fracture. Researchers in clinical nursing must be aware of the differences between *health promotion*, which has an everyday life perspective and *prevention*, which has a medical perspective. They should do research in both perspectives and include well‐documented theoretical concepts; otherwise, the results become blurred.

## 7 PATIENT CONSENT FORM

As this is a systematic mixed methods review, a patient consent is not requested.

## CONFLICT OF INTEREST

The authors declare no conflict of interests.

## AUTHOR CONTRIBUTIONS

Both authors have agreed on the final version of the paper and met at least one of the following criteria (based on those recommended by the ICMJE (https://www.icmje.org/ethical_1author.html):

1. substantial contributions to conception and design, acquisition of data, or analysis and interpretation of data;
2. drafting the article or revising it critically for important intellectual content.
